# Supplementary material for: WordCommentsAnalyzer: A windows software tool for qualitative research
Source: F1000Res. 2018 Sep 4;7:536. Originally published 2018 May 3. [Version 2] doi: 10.12688/f1000research.14819.2 (PMC6137411; doi:10.12688/f1000research.14819.2)
Supplement: Supplementary file 3 [file f1000research-7-17614-s0002.tgz › b2a79496-4ada-47c4-9682-a99ce867836d.zip!/Eight Iranian Health Journals/Yafteh _4.docx]

© Yafteh  . This work is licensed under CC BY 4.0 (https://creativecommons.org/licenses/by/4.0/).

Volume & Issue: دوره 19 - شماره 2

Title: بررسی میزان ایزومرهای ترانس و درصد اسیدهای چرب در روغن‌های حیوانی و دنبه مصرفی در استان لرستان

http://yafte.lums.ac.ir/article-1-2538-fa.html

مقدمه: اسیدهای چرب ترانس جزو چربی‌های مضر برای سلامتی به‌حساب می‌آیند. سازمان غذا و دارو اعلام کرده است که مصرف اسیدهای چرب ترانس باید به کمتر از یک درصد مصرف انرژی برسد. هدف از این مطالعه تعیین میزان اسیدهای چرب با تأکید بر اسیدهای چرب ترانس و اشباع از روغن حیوانی و دنبه بود. مواد و روش­ ها: 10 نمونه روغن حیوانی و 5 نمونه دنبه جهت استری شدن به آزمایشگاه منتقل گردید و با دستگاه کروماتوگرافی گازی با ستون 100 متری و دتکتور FID و حجم تزریق یک میکرولیتر، جهت تعیین پروفایل اسیدهای چرب، نمونه‌ها مورد آنالیز قرار گرفت. یافته ­ها: نتایج نشان داد که مجموع درصد اسیدهای چرب ترانس در روغن‌های حیوانی 5/2 ± 3/4 و مجموع درصد اسیدهای چرب اشباع 7/4 ± 6/47 و در روغن دنبه مجموع درصد اسیدهای چرب ترانس 1/0 ± 3/0 و مجموع درصد اسیدهای چرب اشباع 4/6 ± 7/40 به دست آمد. بحث و نتیجه ­گیری: روغن حیوانی و دنبه گوسفند شامل درصد بالایی از اسیدهای چرب ترانس و اشباع هستند؛ بنابراین ریسک بیماری‌های قلبی عروقی در افراد مصرف کننده بالا می‌رود.

Title: بررسی تأثیر سیتوتوکسیک کورکومین بر تکثیر سلول سرطانی روده بزرگ انسان (HT_29)

http://yafte.lums.ac.ir/article-1-2539-fa.html

مقدمه: سرطان دستگاه گوارش شایع‌ترین نوع در ایران با فراوانی 38 درصد است. خطر ابتلا به این سرطان تحت تأثیر دو عامل محیطی و فاکتورهای ژنتیکی می‌باشد. کورکومین از جمله فیتوکمیکال‌هایی است که در استراتژی chemoprevention در راستای بلوکه کردن، کند کردن و یا برگشت پروسه سرطان‌زایی نقش دارد. مطالعات نشان داده که کورکومین می‌تواند مانع بروز سرطان راست‌روده شده و یا آن را به تأخیر بیندازد. در این پژوهش اثر کورکومین بروی تکثیر سلول‌های سرطانی HT_29 بررسی شد. مواد و روش ­ها: در این پژوهش تجربی رده سلولی HT_29 در محیط DMEM کشت داده شد. اثر غلظت‌های مختلف کورکومین بر روی رشد سلول‌های سرطانی HT_29 با روش MTT بررسی گردید، نوع مرگ سلولی القاء شده توسط رنگ‌آمیزی DAPI سنجش شد. تمامی تجربیات سه بار تکرار شدند و جهت تحلیل داده‌ها از نرم افزار Instate 3 و آزمون آماری one-way ANOVA استفاده گردید. یافته­ ها: بقاء سلولی در غلظت μM 50 کورکومین پس از طی 24 ساعت تیمار، 53 درصد محاسبه شد (IC50). بعد از طی 72 ساعت تیمار، واکوئله شدن و کاهش شدید سلول‌های زنده در محیط کشت مشاهده شد. همچنین، میزان سلول‌های آپوپتوز شده توسط رنگ‌آمیزی DAPI تعیین شد. بحث و نتیجه­ گیری: با توجه به مسیرهای مولکولی دخالت کننده در تکثیر سلولی و توانایی کورکومین در القای پروتئین‌های پروآپوپتوتیک و مهار پروتئین‌های آنتی‌آپوپتوتیک و مسیرهای بقا همچون NF-KB و AKT، می‌توان آن را به‌عنوان یک عامل ضد سرطان معرفی نمود.

Title: بررسی اثربخشی درمان شناختی- رفتاری و مدیریت رفتار بر تعلل ورزی دانشجویان دانشگاه علوم پزشکی لرستان

http://yafte.lums.ac.ir/article-1-2541-fa.html

مقدمه: تحقیقات دو دهه اخیر بیانگر اهمیت و توجه خاص کشورهای مختلف به مسئله تعلل بوده است. این پژوهش با هدف بررسی اثربخشی درمان شناختی-رفتاری و مدیریت-رفتار بر تعلل ورزی دانشجویان دانشگاه علوم پزشکی لرستان صورت گرفته است. مواد و روش­ ها: در این پژوهش روش آزمایشی به کار رفته است که در آن از طرح پیش آزمون، پس آزمون با گروه‌های آزمایش و گواه استفاده شده است. از میان دانشجویان دختر و پسر در سال تحصیلی 94-93، 393 نفر به‌عنوان نمونه آماری پژوهش به‌صورت در دسترس انتخاب شدند. برای انتخاب دانشجویان گروه‌های پژوهشی، ابتدا پرسشنامه تعلل تاکمن برای نمونه‌ای به حجم 393 نفر از دانشجویان اجرا شد و از این میان، تعداد 76 نفر از دانشجویان با توجه به نمرات کسب شده در پرسشنامه تعلل تاکمن به‌عنوان دانشجویان دارای تعلل شناسایی و به دو گروه آزمایش و کنترل تقسیم شدند.   یافته ­ها: یافته‌های این پژوهش نشان داد که میزان شیوع تعلل در کل دانشجویان 33/19 درصد بود. 41/29 درصد از پسران جامعه مورد بررسی، تعللی بودند در حالی که این میزان در دختران 96/14 درصد بود. همچنین نتایج حاکی از تأثیر گذاری دو روش درمان شناختی - رفتاری و مدیریت رفتار بر کاهش تعلل بود. علاوه بر این تفاوت معناداری بین جنسیت و نوع درمان در این پژوهش مشاهده شد. بحث و نتیجه­ گیری: با توجه به نتایج حاصله از این پژوهش، به نظر می‌رسد که تعلل از سازه‌های شناختی تأثیرپذیر است و با توجه به همین مسئله، این دو روش درمانی با تأکید بر سازه‌های شناختی توانستند میزان تعلل را در گروه آزمایش کاهش دهند. همچنین تفاوت روش‌های درمانی در بین دختران و پسران، نشان از تفاوت سازه‌های شناختی متناسب با جنسیت است که به نظر می‌رسد بی‌ارتباط با نقش‌های جنسیتی نباشد.

Title: تأثیر برنامه درمانی مثبت نگر بر بهزیستی روانشناختی زنان دارای کودکان مبتلا به بیش فعالی – نقص توجه

http://yafte.lums.ac.ir/article-1-2542-fa.html

مقدمه: این مطالعه با هدف بررسی اثربخشی برنامه درمانی مثبت­نگر بر بهزیستی روانشناختی زنان دارای کودکان مبتلا به بیش فعالی – نقص توجه انجام شد. مواد و روش­ ها: این مطالعه از نوع شبه­آزمایشی و طرح پیش آزمون - پس آزمون با گروه کنترل بود. نمونه پژوهش شامل 50 مادر دارای کودک مبتلا به بیش­فعالی- نقص توجه در سال 95-94 بود. برای انتخاب نمونه از روش نمونه­گیری سهل­الوصول استفاده شد. شرکت­کنندگان به صورت کاملاً تصادفی در دو گروه 25 نفره تقسیم شدند. این گروه­ها به صورت تصادفی به دو گروه آزمایش و کنترل تقسیم شدند. گروه آزمایش برنامه درمانی مثبت­نگر و گروه کنترل هیچ مداخله­ای دریافت نکرد. بسته درمانی مثبت­نگر طی 6 جلسه آموزشی ارائه شد. قبل و بعد از اجرای آزمایش، همه شرکت­کنندگان به پرسشنامه بهزیستی روانشناختی پاسخ دادند. سه ماه بعد از آزمایش یعنی در پیگیری، جهت بررسی ماندگاری اثرات برنامه، شرکت­کنندگان به پرسشنامه پاسخ دادند. داده­های به دست آمده از طریق آزمون تحلیل کوواریانس چندمتغیره و نرم­افزار آماری SPSS18 تحلیل شدند. یافته ­ها: نتایج این مطالعه نشان داد برنامه مثبت­نگر منجر به تأثیر معناداری در همه 6 مؤلفه بهزیستی روانشناختی شد. لازم به ذکر است که همه نتایج در سطح (05/0>P) و درجات آزادی (48 و 2) معنادار بود و تأثیر آن طی سه ماهه پیگیری پابرجا ماند (05/0>P). بحث و نتیجه­ گیری: با توجه به نتایج به دست آمده برنامه درمانی مثبت­نگر می­تواند در کاهش مشکلات روانشناختی و ارتقای سطح زندگی مؤثر باشد. همچنین این برنامه در ترویج سلامت روان در میان مادران دارای کودکان مبتلا به مشکلات روانشناختی ازجمله بیش­فعالی مؤثر است.

Title: بررسی ژنومی کوکسیلا بورنتی در شیر خام و غیر پاستوریزه گاو در مراکز فروش لبنیات سنتی شهرستان خرم آباد استان لرستان در سال 1394

http://yafte.lums.ac.ir/article-1-2543-fa.html

مقدمه: تب کیو یک بیماری مشترک با انتشار وسیع است که به‌وسیله یک ارگانیسم داخل سلولی اجباری به نام کوکسیلا بورنتی ایجاد می‌شود. شیر خام یا فرآورده­های لبنی تولید شده از شیر غیرپاستوریزه ممکن است حامل کوکسیلا بورنتی عفونت زا باشد. هدف این مطالعه تعیین میزان شیوع کوکسیلا بورنتی در تانک‌های ذخیره شیر خام و غیرپاستوریزه گاو مراکز فروش لبنیات سنتی شهرستان خرم­آباد بود. مواد و روش ­ها: در این مطالعه مقطعی-توصیفی در مجموع 120 نمونه از تانک‌های ذخیره شیر خام و غیرپاستوریزه گاو مراکز فروش لبنیات سنتی شهرستان خرم­آباد طی ماه‌های آبان و آذر سال 94 جمع آوری شد و برای تعیین حضور کوکسیلا بورنتی از روش Nested PCR استفاده شد. یافته­ها: در این بررسی 9 نمونه از 120 نمونه تانک‌های ذخیره شیر خام و غیرپاستوریزه گاو (5/7 درصد) از نظر کوکسیلا بورنتی مثبت شدند. بحث و نتیجه­ گیری: نتایج این مطالعه نشان داد تانک‌های ذخیره شیر خام و غیرپاستوریزه گاو مراکز فروش لبنیات سنتی یک منبع مهم کوکسیلا بورنتی در شهرستان خرم­آباد می­باشد.

Title: بررسی اثرات ضد قارچی گیاه سداب بر کاندیدا آلبیکنس‌های جدا شده از بیماران مبتلا به واژینیت در شرایط invitro و مقایسه با دو آنتی‌بیوتیک، طی دو فصل بهار و زمستان

http://yafte.lums.ac.ir/article-1-2544-fa.html

مقدمه: افزایش مقاومت باکتری‌ها به عوامل ضد باکتری یکی از مشکلات مهم در پزشکی است. گیاهان دارویی در طب سنتی و منابع غنی گیاهی در دسترس، عاملی برای بررسی دقیق‌تر داروهای گیاهی بوده است. برخی از اسانس‌های گیاهی از جمله گیاه سداب دارای اثرات ضد قارچ بوده و از آن می‌توان به‌عنوان عوامل ضد میکروب در درمان عفونت‌ها استفاده کرد. این مطالعه با هدف تعیین اثرات ضد قارچی اسانس حاصل از گیاه سداب انجام شد. مواد و روش‌ها: در این مطالعه تجربی، گیاه سداب از مرکز تحقیقات جهاد کشاورزی استان قم جمع‌آوری شد. سپس اسانس و عصاره گیری شدند. قطر هاله مهاری و حداقل غلظت مهارکننده از رشد و حداقل غلظت کشندگی (MIC، MFC)، 140 جدایه کاندیدا آلبیکنس از زنان مراجعه‌کننده به کلینیک زنان مورد مطالعه قرار گرفت. یافته­ ها: میانگین هاله عدم رشد در روش دیسک دیفیوژن اسانس و عصاره اتانولی سداب در فصل بهار بر روی جدایه‌ها به ترتیب برابر با    mm 5/2±71/19 و mm 9/1±19/16 و در زمستان به ترتیب برابر با mm 7/3±16/21 و mm 2/2±32/18 بود. نتیجه گیری: طبق نتایج این مطالعه، اسانس این گیاه بر رشد قارچ کاندیدا آلبیکنس اثر مهار کنندگی دارد.

Title: تأثیر سه ماه تمرین هوازی با شدت متوسط بر سطوح پلاسمایی IL-6، IL-1β و سطوح مغز زنان 65-50 ساله مبتلا به سندروم متابولیک

http://yafte.lums.ac.ir/article-1-2545-fa.html

مقدمه: افزایش سن، سندروم متابولیک و عوامل التهابی از عوامل مؤثر در کاهش حجم مغز می‌باشند. هدف از این تحقیق بررسی اثرگذاری سه ماه تمرین هوازی با شدت متوسط بر سطوح پلاسمایی IL1β، IL-6 و سطوح مغز زنان 65-50 ساله‌ی مبتلا به سندروم متابولیک می‌باشد. مواد و روش ­ها: 24 زن مبتلا به سندروم متابولیک به‌طور داوطلبانه در این تحقیق شرکت کردند. آزمودنی‌ها به‌طور تصادفی به دو گروه 12 نفره تمرین و کنترل تقسیم شدند. گروه تمرین، در یک برنامه تمرینی هوازی 12 هفته‌ای که هر هفته سه جلسه و هر جلسه شامل سه ست تمرینی و دو ست استراحت بود شرکت کردند. در ابتدا مدت زمان ست تمرینی 8 دقیقه بود که با سپری شدن هر هفته، یک دقیقه به مدت زمان ست­های تمرینی افزوده می­شد. تصویربرداری از سطوح مغز و خون‌گیری برای اندازه‌گیری سطوح مغز، IL-6 و IL1β قبل و سه ماه بعد انجام گرفت. برای تجزیه و تحلیل داده‌ها از روش آماری تی مستقل و تی جفتی استفاده گردید. یافته ­ها: سطوح مغز پس از سه ماه تمرین هوازی تغییر معنی‌داری نکردند. میزان IL-6 و IL1β در اثر تمرین کاهش معنی‌داری یافتند (05/0˂P). بحث و نتیجه­ گیری: این یافته‌ها نشان‌دهنده‌ی تأثیر سه ماه تمرین هوازی با شدت متوسط بر کاهش عوامل التهابی و عدم تأثیر بر سطوح مغز می‌باشد. با توجه به نتایج مذکور این احتمال وجود دارد که تعدیل زمان و شدت تمرین ورزشی موجب تأثیر بر اندازه سطوح مغز گردد.

Title: بررسی و مقایسه اثر 4 هفته تمرینات استقامتی با مصرف داروی فلوکستین بر روی افسردگی و گیرنده‌های بتا 1 آدرنرژیک قلبی در موش‌های نر افسرده نژاد ویستار رت

http://yafte.lums.ac.ir/article-1-2546-fa.html

مقدمه: قرار گرفتن طولانی مدت در معرض اضطراب و افسردگی، میوکاردیوم را در معرض نارسایی قرار می‌دهد. پیام‌های نروهورمونی ناشی از افسردگی از راه گیرنده‌های بتا 1 آدرنرژیک به قلب می‌رسند. هدف از انجام این مطالعه بررسی و مقایسه اثر 4 هفته تمرینات استقامتی با داروی فلوکستین بر روی گیرنده‌های بتا 1 آدرنرژیک قلبی در موش‌های نژاد ویستار رت می‌باشد. مواد و روش ­ها: روش پژوهش حاضر تجربی می‌باشد که در آن 32 سر موش نر نژاد ویستار رت به ‌طور تصادفی به چهار گروه مساوی تقسیم شدند، به هر کدام از رت‌های گروه‌های تمرین و مصرف داروی فلوکستین، 8/0 میلی‌گرم به ازای هر کیلوگرم وزن بدن LPS درون صفاقی تزریق شد. گروه تمرین پروتکل 4 هفته‌ای دویدن روی تریدمیل را به مدت 45 دقیقه در روز انجام دادند. به گروه فلوکستین روزانه 5/0 میلی‌گرم فلوکستین خورانده شد. یافته­ ها: میانگین تراکم گیرنده‌های بتا 1 آدرنرژیک قلبی در گروه تمرین نسبت به گروه افسرده به‌طور معناداری کمتر است (05/0P<). میانگین تراکم گیرنده‌های بتا 1 آدرنرژیک قلبی در گروه تمرین نسبت به گروه فلوکستین معنادار نمی‌باشد. بحث و نتیجه­ گیری: تمرین می‌تواند از طریق کاهش علائم رفتاری افسردگی اثرات مفید را ایجاد کند و با مهار افزایش تراکم گیرنده‌های بتا 1 آدرنرژیک قلبی، موجب کاهش تحریک سمپاتیکی قلب می‌شود.

Title: تأثیر دو سطح مختلف فشار مثبت انتهای بازدمی (PEEP) در میزان بروز آتلکتازی پس از جراحی بای‌پس عروق کرونر

http://yafte.lums.ac.ir/article-1-2547-fa.html

مقدمه: میزان بروز آتلکتازی بعد از عمل جراحی پیوند عروق کرونر بالا می‌باشد. با توجه به تأثیرات پیشنهاد شده‌ی سطوح مختلف PEEP در جلوگیری از بروز این عارضه، هدف از این مطالعه بررسی تأثیرات سطوح مختلف PEEP بعد از عمل جراحی پیوند عروق کرونر بوده است. مواد و روش ­ها: این مطالعه به روش کارآزمایی بالینی انجام گرفت. حجم نمونه در این مطالعه 90 نفر بود. نمونه‌گیری به‌صورت غیر احتمالی متوالی انجام گردید و بیماران به روش بلوک‌بندی طبقه‌ای به‌طور تصادفی در دو گروه مداخله و کنترل (هر گروه 45 نفر) قرار گرفتند. در گروه کنترل بیماران پس از عمل جراحی، به‌محض ورود به بخش مراقبت‌های ویژه تا زمان اکستیوب شدن PEEP به میزان 5 سانتی‌متر آب دریافت می‌کردند و در گروه مداخله بیماران پس از ورود به بخش مراقبت‌های ویژه به‌طور متوسط به مدت 4 ساعت PEEP به میزان 10 سانتی‌متر آب دریافت می‌کردند و سپس تا زمان اکستیوب شدن PEEP به میزان 5 سانتی‌متر آب دریافت می‌کردند. برای تشخیص نهایی آتلکتازی شش ساعت بعد از اکستیوب شدن، از رادیوگرافی قفسه سینه، درصد اشباع اکسیژن شریانی و درجه حرارت بدن استفاده شد. برای بررسی‌های آماری از آزمون‌های مجذور کای و تی مستقل استفاده شد. یافته ­ها: بر اساس یافته‌های پژوهش میزان بروز آتلکتازی در گروه مداخله 7 نفر (6/15 درصد) و در گروه کنترل 16 نفر (6/35 درصد) بود که از لحاظ آماری این تفاوت معنادار بود (03/0=P). میانگین درصد اشباع اکسیژن شریانی در گروه مداخله نسبت به گروه کنترل بالاتر بود (48/1±31/94 در مقابل 16/1±66/93) و این تفاوت از نظر آماری معنادار بود (025/0=P). بحث و نتیجه­ گیری: این مطالعه نشان داد که استفاده از PEEP به میزان 10 سانتی‌متر آب بعد از عمل جراحی پیوند عروق کرونر می‌تواند باعث کاهش میزان بروز آتلکتازی بعد از عمل شود، بنابراین پیشنهاد می‌گردد که از این روش در بخش مراقبت‌های ویژه برای کاهش میزان بروز آتلکتازی استفاده شود.

Title: بررسی تأثیر طرح تحول نظام سلامت بر شاخص‌های عملکردی مراکز بیمارستانی تحت پوشش دانشگاه علوم پزشکی لرستان

http://yafte.lums.ac.ir/article-1-2549-fa.html

مقدمه: نظام‌های سلامت به‌منظور تطابق با شرایط در حال تغییر و پاسخگویی به نیازهای جامعه، نیازمند تغییرات و اصلاحات مداوم در ساختار و عملکرد و متعاقب آن نیازمند ارزشیابی شاخص­هایشان هستند. لذا هدف این مطالعه بررسی تأثیر طرح تحول نظام سلامت بر شاخص‌های عملکردی بود. مواد و روش ­ها: پژوهش حاضر مطالعه‌ای توصیفی تحلیلی بود که به‌صورت مقطعی و با استفاده از داده‌های سال‌های 1392 تا 1394 یعنی سال­های قبل و بعد از اجرای طرح تحول نظام سلامت انجام گرفت. جامعه آماری پژوهش کلیه بیمارستان‌های دانشگاه علوم پزشکی لرستان (14 بیمارستان) بود. برای آنالیز داده­ها از آزمون­های آماری تی زوجی و ویلکاکسون با نرم‌افزار SPSS نسخه 16 استفاده شد. یافته­ ها: بعد از طرح تحول سلامت همه شاخص­ها نسبت به قبل وضعیت بهتری داشته‌اند و این ارتقاء در تمام موارد معنی‌دار بود. فقط متوسط اقامت بیمار بعد از طرح تحول افزایش اندکی (5/0%) داشت که البته این افزایش نیز از نظر آماری معنی‌دار نبود (653/0=P). قبل از اجری این طرح، اختلاف میانگین فقط برای شاخص ضریب اشغال تخت بین مراکز آموزشی و غیر آموزشی معنی‌دار بود (05/0P <)، اما بعد از طرح، اختلاف میانگین برای همه شاخص‌ها جز شاخص متوسط طول اقامت (038/0=P)، معنی‌دار بود. بحث و نتیجه ­گیری: طرح تحول در نظام سلامت، سبب تغییر مثبت در شاخص‌های عملکردی بیمارستان شده است. لذا در عین توجه به بهبود مستمر روند کنونی، تداوم در اجرای طرح مذکور بر اساس نتایج حاصل از این مطالعه توصیه می‌گردد.

Title: نقش گیرنده‌های موسکارینی بر انقباضات ایلئوم جدا شده موش صحرایی نر بالغ

http://yafte.lums.ac.ir/article-1-2550-fa.html

مقدمه: اثرات سیستم کولینرژیک از طریق دو نوع گیرنده نیکوتینی و موسکارینی اعمال می‌گردد. با توجه به وجود گیرنده‌های موسکارینی در ایلئوم موش صحرایی، در این مطالعه، اثر کارباکول (آگونیست گیرنده موسکارینی) و اسکوپولامین (آنتاگونیست گیرنده موسکارینی) بر انقباض ایلئوم ناشی از KCl در موش صحرایی نر بالغ مورد بررسی قرار گرفت. مواد و روش ­ها: بخش انتهایی ایلئوم موش صحرایی نژاد ویستار جدا شد و انقباضات آن تحت یک گرم کشش و دمای 37 درجه سانتی‌گراد، در حمام بافت حاوی محلول تایرود به روش ایزوتونیک ثبت شد. بافت ایلئوم به‌صورت جداگانه در گروه اول تحت تأثیر KCl سپس کارباکول، گروه دوم تحت تأثیر KCl سپس اسکوپولامین، گروه سوم تحت تأثیر اسکوپولامین به مدت 30 دقیقه سپس KCl، گروه چهارم تحت تأثیر اسکوپولامین با غلظت 3- 10 مولار به مدت 30 دقیقه سپس کارباکول با غلظت 2-10 میلی‌گرم بر میلی‌لیتر قرار گرفت. یافته ­ها: تفاوت معنی‌داری بین غلظت صفر و غلظت‌های (mg/ml2-10، 3-10، 4-10) کارباکول وجود داشت (001/0P<). تفاوت معنی‌داری بین غلظت صفر و غلظت M 3-10 اسکوپولامین (01/0P<) و نیز مقادیر M 4-10، 6-10  وجود داشت (001/0P<). تفاوت معنی‌داری بین غلظت صفر و غلظت M 3-10 اسکوپولامین (01/0P<) و نیز مقادیر  M 4-10 (001/0P<) و M 6-10 (05/0P<) زمانی که بافت به مدت 30 دقیقه در معرض اسکوپولامین قرار گرفت، وجود داشت. بحث و نتیجه ­گیری: این مطالعه نشان داد که غلظت‌های مختلف کارباکول انقباض ناشی از کلرور پتاسیم را به‌صورت وابسته به غلظت تقویت می‌کند و با کلرور پتاسیم اثر سینرژیسم دارد. همچنین غلظت‌های مختلف اسکوپولامین انقباض ناشی از کلرور پتاسیم را به‌صورت وابسته به غلظت کاهش می‌دهد.

Title: ارزیابی سایتو‌‌توکسیسیتی و اثرات ضد پلاسمودیومی عصاره‌های اتانولی و دی‌کلرومتان بره موم چهار منطقه مختلف ایران

http://yafte.lums.ac.ir/article-1-2551-fa.html

مقدمه: ظهور مقاومت دارویی در برابر انگل مالاریا یکی از مشکلات کنترل و حذف مالاریا در جهان محسوب می‌شود. لذا در این تحقیق به ‌منظور غلبه بر مقاومت دارویی بره موم چهار منطقه متفاوت ایران از نظر سایتوتوکسیسیتی و خواص ضد پلاسمودیومی بررسی شد. مواد و روش ­ها: بره موم‌های ایران از چهار منطقه مختلف جمع‌آوری و با حلال‌های اتانول 70% و دی‌کلرومتان عصاره‌گیری شدند. سایتوتوکسی عصاره‌های بره موم با روش MTT بر روی سلول فیبروبلاست L929 بررسی و اثرات ضد پلاسمودیومی عصاره‌های مختلف بره موم در شرایط درون تنی بر روی موش‌های نژاد BALB/c بررسی شد. یافته­ ها: عصاره اتانولی و دی‌کلرومتان بره موم‌های چهار منطقه مختلف ایران در دوزهای µg/ml 50، 100 و 200 غیر سمی بودند. بیشترین درصد مهار رشد انگل Plasmodium berghei با 71 و 65 درصد به ترتیب مربوط به عصاره اتانولی و دی‌کلرومتان بره موم منطقه مرادبیگ بود. بحث و نتیجه ­گیری: با توجه به وجود مقاومت پلاسمودیوم فالسیپاروم به داروهای رایج و با توجه به خطرات بروز اپیدمی ناشی از این مقاومت دارویی و اهمیت یافتن و توسعه داروهای مؤثر، ارزان و بی‌خطر در برنامه‌های کنترل و حذف بیماری مالاریا، پیشنهاد می‌شود خاصیت ضد پلاسمودیومی بره موم‌های ایران در تحقیقات تکمیلی مورد بررسی بیشتری قرار گیرند.

Title: بررسی اثر پپتید آنتاگونیست فاکتور رشد فیبروبلاستی بر رشد تومور پستان موشی ، سطح سرمی اینترلوکین 8 و فاکتور نکروز دهنده تومور آلفا

http://yafte.lums.ac.ir/article-1-2556-fa.html

مقدمه: سرطان پستان امروزه مهم‌ترین عامل تهدیدکننده سلامتی در زنان است. در اکثر سرطان­ها علت اصلی شکست در درمان، متاستاز سلول‌های سرطانی است. جراحی و رادیوتراپی در درمان تومورهای موضعی کاربرد مناسب دارد اما در متاستازهای سرطانی کارایی لازم را نداشته و در چنین مواردی روش درمان اغلب بر پایه­ی شیمی‌درمانی استوار است .به دلیل آثار جانبی داروهای شیمی‌درمانی و کمتر بودن این اثر در پپتیدها، در حال حاضر پپتید درمانی در دنیا رواج یافته است. لذا هدف از تحقیق حاضر بررسی اثر پپتید آنتاگونیست bFGF طراحی شده بر مهار رشد تومور در مدل سرطان پستان متاستاتیک 4T1 و میزان سطح سرمی IL8 و TNFα می­باشد. مواد و روش ­ها: در این تحقیق از موش‌های نژاد BALB/C ماده­ی 7-5 هفته­ای استفاده شد. تومور به روش پیوند تومور4T1 ایجاد شد. پس از گذشت دو هفته و مناسب شدن اندازه­ی تومورها، تیمار آغاز شد و با پپتید­ طراحی شده در سه دوز انتخابی به صورت درون صفاقی و به مدت 14 روز تزریق صورت گرفت. در هر بار تزریق از کنترل مثبت و منفی نیز استفاده شده است. به موش‌های گروه کنترل منفی PBS و به موش‌های گروه کنترل مثبت داروی شیمی‌درمانی دوکسوروبیسین تزریق گردید. سایز تومورها به صورت یک روز در میان با استفاده از کولیس دیجیتال اندازه­گیری شد و حجم تومور با استفاده از فرمول طول در عرض 2 در 5/0 محاسبه شد. بعد از خونگیری از چشم موش‌ها و جداسازی پلاسما میزان تولید IL8 و TNFα به روش الایزا انجام شد. یافته­ ها: با استفاده از آزمون One Way ANOVA با کمک نرم‌افزار SPSS تجزیه و تحلیل آماری صورت گرفت و با توجه به 05/0P< مشخص شد که تزریق پپتید بر روی کاهش و یا مهار رشد تومور اثر داشته و اختلاف معناداری بین گروه کنترل منفی با گروه کنترل مثبت و گروه تیمار شده با پپتید وجود دارد.میزان سطح سرمی IL8 و TNFαدر تومورهایی که پپتید دریافت کرده اند نسبت به گروه کنترل منفی اختلاف معناداری دارد. بحث و نتیجه­ گیری: نتایج این تحقیق نشان داد که پپتید ضد رگزایی به طور موثری موجب مهار رشد تومور پستان شده و میزان سطح سرمی IL8 و TNFαدر تومورهایی که پپتید دریافت کرده اند نسبت به گروه کنترل منفی اختلاف معناداری دارد.
